# Scenario Screen: A Dynamic and Context Dependent P300 Stimulator Screen Aimed at Wheelchair Navigation Control

**DOI:** 10.1155/2018/7108906

**Published:** 2018-02-14

**Authors:** Omar Piña-Ramirez, Raquel Valdes-Cristerna, Oscar Yanez-Suarez

**Affiliations:** Neuroimaging Research Laboratory, Electrical Engineering Department, Universidad Autonoma Metropolitana Unidad Iztapalapa, Av. San Rafael Atlixco 186, Col. Vicentina, Iztapalapa, 09340 Ciudad de México, Mexico

## Abstract

P300 spellers have been widely modified to implement nonspelling tasks. In this work, we propose a “scenario” stimulation screen that is a P300 speller variation to command a wheelchair. Our approach utilized a stimulation screen with an image background (scenario snapshot for a wheelchair) and stimulation markers arranged asymmetrically over relevant landmarks, such as suitable paths, doors, windows, and wall signs. Other scenario stimulation screen features were green/blue stimulation marker color scheme, variable Interstimulus Interval, single marker stimulus mode, and optimized stimulus sequence generator. Eighteen able-bodied subjects participated in the experiment; 78% had no experience in BCI usage. A waveform feature analysis and a Mann–Whitney *U* test performed over the pairs of target and nontarget coherent averages confirmed that 94% of the subjects elicit P300 (*p* < .005) on this modified stimulator. Least Absolute Shrinkage and Selection Operator optimization and Linear Discriminant Analysis were utilized for the automatic detection of P300. For evaluation with unseen data, target detection was computed (median sensitivity = 1.00 (0.78–1.00)), together with nontarget discrimination (median specificity = 1.00 (0.98–1.00)). The scenario screen adequately elicits P300 and seems suitable for commanding a wheelchair even when users have no previous experience on the BCI spelling task.

## 1. Introduction

Noninvasive Brain-Computer Interfaces (BCI) are systems that translate the electrical brain activity measured through Electroencephalography (EEG) into executable commands for any enabled device [1]. BCI were developed to augment the communication and environment interaction possibilities for patients with severe motor disabilities such as amyotrophic lateral sclerosis, spinal cord injury, or locked-in syndrome [2–4].

The P300 speller is a computer based dictation device controlled through the P300 Event-Related Potential (ERP) which is a cognitive brain response elicited by the stimulation-dependent (synchronous)* oddball* paradigm [1, 2, 4]. The P300 speller is one of the most used BCI, whose conventional stimulation screen has solid-homogeneous black color background with a symmetric and homogeneous matrix arrangement of 6 × 6 stimulation markers: 26 English alphabet characters, nine decimal digits, and the underscore for blank space. The stimulation consists of a random sequence of marker flashes on single or multimarker mode. Then, when the users perceive a flash stimulus on the symbol to which they are focused on, a P300 ERP is elicited [2, 4]. Finally, the spelling task process relates an automatic detection of the P300 to the letter that generates it [2, 4–6].

P300 speller variations have included matrix size, marker arrangement, marker types, stimulus sequence, and stimulus presentation and have been tested to increase the information transfer rate and the detection rate and even to perform nonspelling tasks. A summary of these variations is described next, to contextualize the stimulation screen presented in this work.

Sellers et al. [7] used a 3 × 3 P300 speller matrix and estimated the optimal Interstimulus Interval (ISI) and Stimulus Duration (SD) to achieve detection and transfer rates similar to the 6 × 6 conventional size speller. Colwell et al. [8], Jin et al. [9–11], and Shi et al. [12] utilized rectangular speller matrices of 9 × 8, 7 × 12, and 6 × 12, respectively. Regarding the stimulus presentation, some successful approaches were the submatrix stimulation sequences [13], disperse multimarker stimulation [9–11, 14], blue/green color scheme of stimulation markers for nonflashing/flashing states [15–17], and face paradigm [9, 18–20]. Furthermore, all these variants had a solid black color as background. In respect of P300 detection performances, high rates were reached for all the variations mentioned above.

The geometric variation named* Geospell* [21] displayed screen sequences instead of flash events. Each screen had six characters in a circular arrangement, whose center has a cross symbol where the subjects fix their attention. Each group of six characters presented corresponded to the conventional 6 × 6 matrix rows and columns. Another geometric variation was the lateral single character speller, proposed by Pires et al. [5], which rearranged the stimulation markers laterally on the screen preserving a regular geometry. Its stimulus was presented in single marker mode that pseudorandomly alternates between right and left. Both geometric variations demonstrated high target detection and transfer rates. For all these speller variants, backgrounds remain solid black color.

An approach of the Internet browsing task using a P300 speller-like variation was reported by Mugler et al. [22]. A pair of monitors were used, one for stimulus presentation and the other for web browser display. A homogeneous 8 × 8 stimulation matrix in row/column mode was used. On the other hand, Yu et al. [23] implemented an oddball paradigm to move the mouse cursor into the web browser. Additionally, Halder et al. [18] also used two monitors, one of them for face paradigm stimulus presentation of a P300 speller that was reshaped according to the number of available browsing commands, each represented with alphabet characters. On another monitor, the web browsing task was performed.

Carabalona et al. [24] and Aloise et al. [25] adapted the conventional speller to control a domotic system. Both cases used icons as stimulation markers instead of alphabet letters. However, they used the typical flashing and background color schemes. A different stimulation screen for domotics was proposed by Hoffmann et al. [26] where RGB images were placed over the solid white background as stimulation markers arranged on a rectangular 3 × 2 matrix. Meanwhile, the smart house control proposed by Corralejo et al. [27] used RGB images and their corresponding text as stimulation markers; the background was solid bright green; the text and its outline were, respectively, white and black. In addition, Ganin et al. [28, 29] demonstrated that moving and consequently asymmetrically arranged stimulation markers elicited P300 on a three-trial paradigm obtaining an average hit rate of 0.8. This paradigm was used in [29] to control a puzzle game, whose stimulation markers are circular puzzle pieces, each labeled with a Cyrillic character.

P300-based wheelchair navigation consists of two components: the P300-based selection of the displacement command and the robotic system that performs the navigation. In this context, there are, essentially, two schemes for selecting the navigation routes: selecting a direction in which a fixed displacement will occur (with stimulation sequences after each fixed displacement) or selecting a destination from some sort of local or global map (with stimulation sequences after attaining each selected destination). Rebsamen et al. [35, 36] introduced a P300 3 × 3 stimulation matrix that had names of apartment rooms and appliances like TV, lights, bed, and so on, as stimulation markers. Its robotic navigation system managed to reach the localization with no additional stimulation sequences while the wheelchair was in transit. All the paths and destinations were predefined. That is to say, the approach implemented stimulation sequences favoring long and complex navigation routes on known scenarios. On the other hand, two approaches introduced a stimulation screen that utilized image-arrows as stimulation markers over a solid color background, emulating a joystick wheelchair. Lopes et al. [34] presented the stimulation in single marker mode for commanding an actual robotic navigation of the wheelchair. Differently, Gentiletti et al. [33] utilized row/column mode in a virtual navigation controller. Both works implemented stimulation sequences after fixed displacements, since each selection corresponds to a short piece of route. Though the joystick stimulators enable navigation on unknown scenarios, a high amount of selections is needed to perform long pathways; therefore, the participants fatigue and lower P300 detection rates were obtained [38, 39]. Notwithstanding, Gentiletti et al. [33] incorporated some kind of destination-oriented stimulation by including a control automata for* wall-following*. Other three approaches incorporated the current information of the navigation scenario into the stimulation screen. Notably, Iturrate et al. [32] integrated a real-time rendered virtual representation of the actual navigation scenario into the stimulation screen, where floor, walls, and obstacles were distinguishable. The stimulation markers for navigation were presented as dots in an arrangement that indicates distance and direction using a polar coordinate system grid centered at the wheelchair. Additional stimulation markers for auxiliary commands were shown on a menu bar; the stimulus presentation was in row/column stimulation mode with a fixed displacement sequence scheme.

Similarly, Escolano et al. [30] implemented a polar grid and menu bar commands, but using augmented reality. That is to say, the virtual representation of the scenario screen, as in [32], was set to low opacity and overlaid to its real counterpart. Thus, a mixed image was presented as stimulation screen whose markers performed row/column stimulation mode with stimulation sequences for fixed displacement according to which a teleoperated robot was controlled. In order to change the robot field of view, a stimulation screen with a gray scale image as background and a square grid of circles as stimulation markers was implemented. In another study, Escolano et al. [30] reported another P300–based approach for commanding a teleoperated robot. It used a gray scale image of 60 × 60 pixels resolution. The stimulation markers were six icons split in two menu bars, and six circles for navigation points which were arranged in a rectangular grid of 2 × 3. All of them were in fixed positions over the screen performing row/column stimulation for fixed displacement.

This work introduces the* scenario screen* stimulator, which is a P300 speller variant. The scenario screen has an image background that is a snapshot of the wheelchair perspective and whose stimulation markers are located over relevant landmarks. To contrast the scenario screen features with other reported P300-based stimulation screens for navigation tasks, a comparative chart is shown in Table 1. Except for the scenario screen, all the other approaches utilized symmetric stimulation markers arrangements and were focused on the evaluation of the wheelchair navigation task. Although some studies indirectly reflected that an image background does not impact the target detection, further analysis in terms of the P300 elicitation, stimulus presentation, and classifier adaptation is needed.

## 2. Materials and Methods

### 2.1. Overview

**Figure 1:**
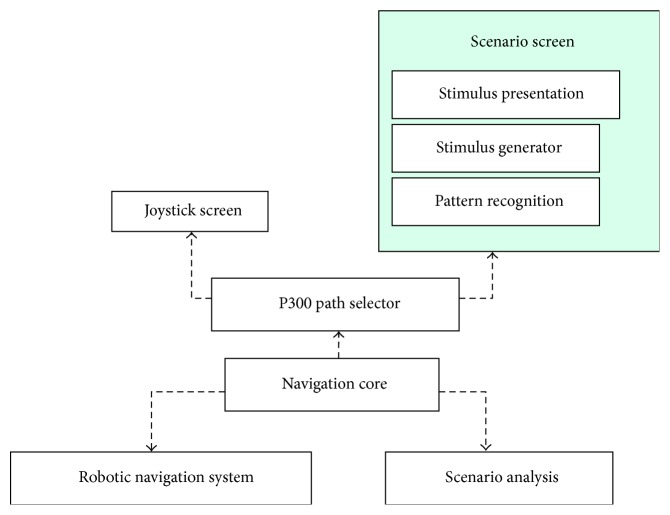
Schematic diagram of a robotic wheelchair navigation system where the scenario screen is utilized for the navigation command. See text for the description.

A scheme of the wheelchair navigation system within which the scenario screen is incorporated is shown in Figure 1. It is briefly described in the following, for the sake of establishing the role of the stimulator that is the subject of this document.

The Navigation Core component coordinates the workflow and the datapath throughout all the components of the system. The Scenario Analysis component takes photographs of the current frontal wheelchair perspective which is analyzed by computer vision methods searching for landmarks, in particular navigation paths, doors, windows, and wall signals. The P300 Path Selector component produces two different stimulation screens. The first is the proper scenario screen, which is the default stimulator screen. Its stimulation markers are located over the most relevant landmarks according to Scenario Analysis component results; the second, for unexpected, finer-control situations, a P300 joystick-like variant is displayed instead. The robotic navigation system controls all the devices and sensors mounted on the wheelchair for driving it safely to its destination, either with the destination-based selections from the scenario screen or following fixed displacement commands from the joystick screen.

**Figure 2:**
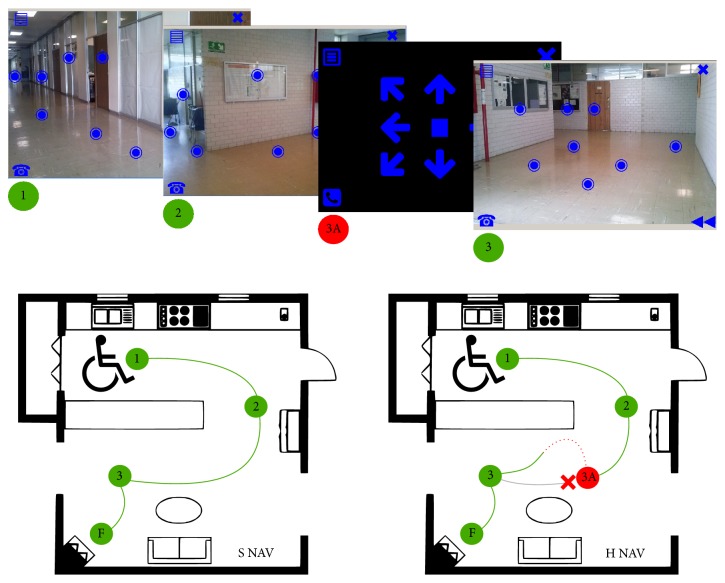
Scenario screen navigation scheme. Each green circle represents where the wheelchair stops to generate the scenario screen. The green lines are the paths followed by the wheelchair. The red point (3A) is where an obstacle interrupts the navigation. The dotted red line represents a fixed displacement commanded navigation.

Figure 2 exemplifies two realizations for navigating from point 1 to point F, based on the scheme detailed above. The navigation based on the scenario screen (S NAV) only performs stimulation sequences at points 1, 2, and 3 (destination-based sequences), where stimulation marker localizations vary according to the image background. For H NAV navigation, the scenario screens and routes are the same as in S NAV. Nonetheless, an eventuality (obstacle) occurs at point 3A (between 2 and 3) and then the joystick-like screen is shown. Several fixed displacement stimulation sequences are run with this screen to sort the obstacle, and the previous navigation path is resumed to reach point 3 where scenario screen is displayed again.

The robotic navigation system is currently under active development in our research team.

### 2.2. Scenario Screen Development

Figure 1 shows the three components of the scenario screen: stimulation screen, stimulus generator, and pattern recognition system. In this section, the first two components are detailed.

#### 2.2.1. Design and Implementation

**Figure 3:**
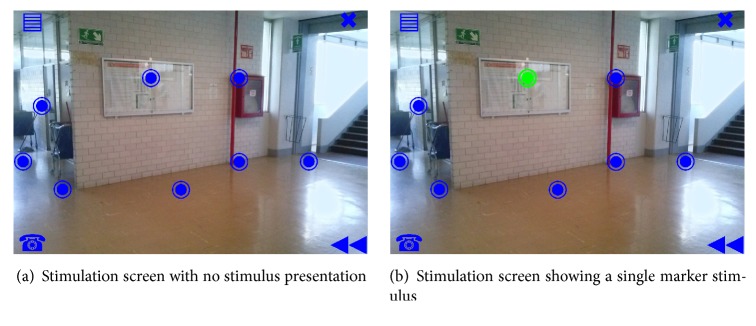
Scenario screen. In this instance, eight markers are arranged in landmarks as follows: five on the floor, two on wall signs, and one on a door. Four markers placed at corners were reserved for other tasks. The background is an unprocessed RGB image of a corridor in the research building.

The scenario screen is a P300 speller variant that enables users to select a destination of navigation.  Figure 3 shows the scenario screen evaluated in this work. As it can be seen, two were the major variations implemented. First, it incorporates the current scene information into the stimulation screen through an RGB image background that depicts the wheelchair navigation perspective, instead of using the conventional solid color background or a virtual-real combination. In this case the image corresponded to an RGB 1024 × 825 pixel snapshot of the research building hall at the University, photographed with a 2.2 Mpx mobile phone camera, with no preprocessing. Second, only twelve stimulation markers, grouped into navigation and auxiliary, replaced the original 36 characters. Using the Unicode fisheye character, the eight navigation markers were located over the most relevant landmarks, in this case: three markers for the main corridor, two for the secondary corridor, one for the door, and two for the wall signals. The four auxiliary markers also used Unicode characters, each having a static placement on screen corners. Their placement, meaning, and encoding are as follows: top left: “open menu” task; bottom left: “call to a predefined contact”; top right: “close stimulation screen”; bottom right: “return to previous wheelchair location.” For this work's purposes, all the stimulation markers were manually located. The scenario screen was implemented by modifying the Native OpenViBE P300 speller GUI [40]. The 6 × 6 stimulation matrix was enlarged to 9 × 9. Except for the twelve stimulation markers, the 81 available labels of the enlarged matrix were not displayed and all the label backgrounds were set to zero opacity to make GUI canvas visible, with the image background set within [41]. Thus, users see the stimulation screen as shown in Figure 3.

#### 2.2.2. Stimulus Presentation

Nonflashing and flashing color scheme were blue/green with RGB encoding (0,0, 255) and (0,255,0), respectively, as used in [15, 16]. The font size of the twelve stimulation markers was fixed to 120 points for both states. These customizations were made with OpenViBE designer tool v0.14 [40].

#### 2.2.3. Sequence Generator Development

A new stimulus/target sequence generator engine was developed to deal with the shortcomings of its OpenViBE P300 speller stimulus generator counterpart. The stimulator engine developed implemented the following features: pseudorandom ISI, single marker stimulus mode, and a strategy to avoid two consecutive stimulations of the same marker. In order to perform the stimulus presentation, this engine interfaced with OpenViBE designer core through proper native OpenViBE ID directives. Furthermore, the stimulus generation was implemented with two modules: the first of them was a Python script that computed and stored a large set of paired stimulus-timing sequences, while the second module was a lua script executed by OpenViBE designer during the acquisition process; it loads the stored stimulation sequences and implements the scenario screen stimulation paradigm described in the following sections.  Figure 3(b) shows the single marker stimuli as seen by users.

### 2.3. Scenario Screen Paradigm

This section describes the paradigm to evaluate the ERP elicitation capabilities of scenario screen. EEG acquisition setup and subject selection are also detailed.

#### 2.3.1. Scenario Screen Stimulation Paradigm

**Figure 4:**
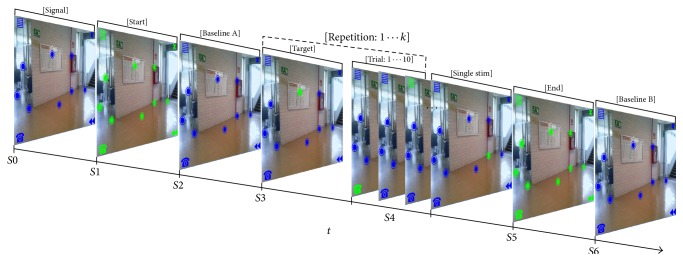
Stimulation sequence repetition. The sequences outline how each stimulation screen state is shown to the user. *Si* represents de *i*th state and *k* = {3,5, 6,6} the number of repetitions.

Following the OpenViBE nomenclature, a trial was a random flashing sequence of the 12 available scenario screen stimulation markers. All flash durations were 0.125 s. The ISI produced by a stimulus generator took values among 0.12, 0.14, 0.16, 0.18, 0.20, and 0.22 s. Intertrial Interval (ITI) was set to 0.5 s. In this form, a repetition (Figure 4: S4) was the sequence of 10 trials performed to select one marker. All repetitions run in copy mode indicate the target to subject by blinking it five times at 1.0 Hz (Figure 4: S3). Finally the Interrepetition Interval was set to 5.0 s.

**Figure 5:**
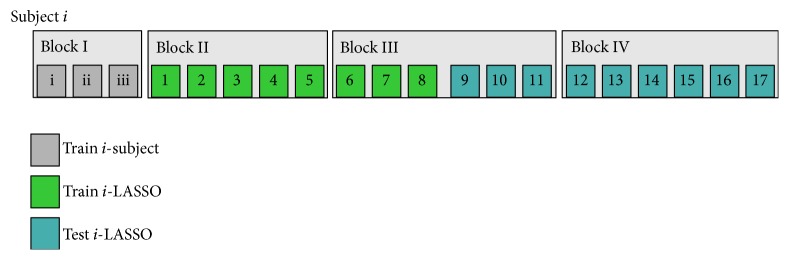
The four blocks which comprise the acquisition session for the *i*th subject. Each color filled square represents a repetition.

The initial stimulation screen state is shown in Figure 4: S0. To indicate task starting time, the twelve available stimulation markers were turned to green during 5 s (Figure 4: S1) after which they all returned to blue. 20 s baseline EEG was acquired (Figure 4: S2); previously the subjects were instructed to stay calm and to remain with open eyes but blink and breathe normally. Subjects performed four blocks of *k* = {3,5, 6,6} repetitions as Figures 5 and 4: S3 and Figure 4: S4 show. Once the last repetition of the current block was accomplished, the block's ending indication was shown with 5 s of green markers (Figure 4: S5). Then, another 20 s of baseline EEG was recorded (Figure 4: S6).

Block order was preserved for all subjects. Block I was used to train subjects on the correct usage of the scenario screen; thus the data from this block was not considered for analysis purposes. Data from Blocks II, III, and IV provided a set of 17 repetitions for analysis; see Figure 5. Given that all these 17 repetitions were performed in copy mode, a pseudorandom strategy that only allowed each of the available markers to be selected as the target once or twice was implemented in the developed stimulus generator engine. Finally, resting time within blocks was from 30 to 60 s during which the subjects answered the questionnaire previously detailed.

#### 2.3.2. Experimental Setup

EEG recordings utilized eight gold surface passive electrodes (Fz, C3, Cz, C4, P3, Pz, P4, and Oz) fixed to an extended 10–20 g.tec cap (Guger Technologies™). The reference was the joint A1-A2 connected through a pair of gold earlobe clip electrodes. A gold cup electrode at right mastoid was the ground. In all cases the electrode-gel–skin impedance was lower than 10 k*Ω* [33, 42]. A g.USBamp biosignal amplifier (Guger Technologies) was used for the recordings with the following configuration: 512.0 Hz sample rate, 8th-order 0.1–30.0 Hz passband, and 4th-order 58.0–62.0 Hz notch Butterworth hardware filters. For this configuration, the OpenViBE acquisition server v0.14 tool was used [40].

Nineteen able-bodied subjects (20–35 years) participated in the experiment; 15 of them had no previous experience in BCI usage. All subjects gave their informed consent by signing the corresponding form and were asked to have at least six hours of sleep the night before the study. Exclusion criteria were intake of stimulant or depressive substances within the six previous hours to the study, any pathological or psychological condition, and light flashing sensitivity. Subjects sat in front of a 22′′ LCD in a comfortable position. They were asked to always blink normally and move only if it was necessary. Eyes–LCD distance was approximately 1.2 m. They were instructed on how to use the scenario screen and also to count mentally how many times the target flashed [42].

### 2.4. Data Analysis

Data from each subject was separately analyzed in two stages:

- Global P300 elicitation: the 17 target coherent averages of each subject were compared with their corresponding nontarget counterpart. A waveform analysis of target averages searching P300 features and statistical tests of difference between target and nontarget averages were performed.
- Evaluation of the automatic target/nontarget detection: a classifier was trained for each subject for automatic labeling of the target and nontarget stimulation markers. Trained classifiers labeled unseen data and their performances metrics were reported, namely, sensitivity, specificity, and accuracy. The behavior of these metrics was analyzed with respect to the number of trials used to make the decisions; group and individual analysis are reported.

#### 2.4.1. Preprocessing Data

The same offline preprocessing was applied for the two analysis stages. The EEG signals were filtered with a 40th-order 1.5–10.0 Hz FIR passband filter designed with a Chebyshev window. Event-related epochs of 307 samples (600 ms) were extracted from signals. Each epoch was detrended and normalized to zero mean and unit variance.

#### 2.4.2. Global P300 Elicitation

Each subject's target and nontarget coherent grand averages were computed using the full data set (Blocks II, III, and IV) combining the eight electrode epochs. A waveform analysis was performed on the target averages observing for the occurrence of characteristic P300 features, such as the peak within 150–450 ms, in addition to N100, N200, and P200 ERP. Two tailed corrected Mann–Whitney *U* tests (MWUT) were computed for each pair in order to test whether there were differences between target average and its nontarget counterpart. Those statistical comparisons were computed using a window spanning 150 to 450 ms after stimulus onset, averages where typically the P300 appears [5, 43–45]; two tailed corrected *p* values are reported.

#### 2.4.3. Automatic Target Detection: Classifier Training

Feature vectors **x** were obtained directly from the preprocessed epochs; each of them were decimated by a factor of two and concatenated giving vectors of 1228 features (*d*). These vectors were arranged by temporal repetition occurrence, stimulation marker ID, and temporal trial occurrence.

Let {(**x**_*i*_, *y*_*i*_) : **x**_*i*_ ∈ *ℝ*^*d*^, *y*_*i*_ ∈ {−1,1}} be the training data set, where **x**_*i*_ is the *i*th feature vector and *y*_*i*_ is its corresponding target or nontarget label. The* Least Absolute Shrinkage and Selection Operator* (LASSO) is a Linear Discriminant Analysis (LDA) variation that uses the *l*_1_–norm as regularizer:

(1) βLASSO=arg⁡minβ⁡∑i=1Nyi−β0−∑j=1dβjxij2+λ∑j=1dβj.

The sparsity parameter *λ* needs to be adjusted; however, the optimal *λ* can be estimated through a cross-validation process. LASSO approach is preferred since some *β* components become equal to zero according to the sparsity parameter *λ* ∈ 0,1]. Thus, it is also considered a feature selection method [46–48].

Let the reduced feature vector **x** correspond to the nonzero features of **w** = *β*^LASSO^. The score denoted by *s*(**x**) is computed with the discrimination function (see (2)) [49–51] that measures the feature vector membership to the target or nontarget class, as a distance to the regression hyperplane:

(2) $$\begin{array}{c} s\left(\;x\;\right)=w^{T}x+b. \end{array}$$

A LASSO-LDA was trained for each subject using the data from their corresponding Blocks II and III as Figure 5 shows. No class balancing method was utilized (Python machine learning library: scikit–learn LASSO through LARS method and sparsity parameter *λ* was estimated by cross-validation [52]). Target labeling was performed in two stages: (1) to score the feature vectors **x** (see (2)) of a given repetition and (2) to select the target through a voting scheme based on the score accumulation (see (3)) [49, 51, 53]:

(3) $$\begin{array}{c} target=\arg\max\left\{\;\overset{L}{\underset{l=1}{\sum }}w^{T}x_{\left(m,l\right)}:m\in \left\{\;1,\ldots ,12\;\right\}\;\right\}, \end{array}$$

where **x**_(*m*, *l*)_ is the feature vector associated with the *m*th stimulation marker on the *l*th trial over a given repetition. Note that the bias *b* of (2) was not considered for calculation since it is constant across all feature vectors [50, 51].

#### 2.4.4. Automatic Target Detection: Classifier Performance Evaluation

Unseen epochs from the last nine repetitions of Blocks III and IV were labeled as target and nontarget via trained LASSO-LDA and voting scheme described above. Three were the metrics for classifier evaluation:* sensitivity*, also known as true positive rate, hit rate, or recall,* specificity* or true negative rate, and* accuracy* or the fraction of data correctly classified [53]. These metrics were reported for user sample and individually with respect to the number of trials scored (*l*).

## 3. Results and Discussion

### 3.1. Stimulation Screen Design and Development

A seven-item questionnaire was answered by users after the stimulation session. Closed-form yes/no questions asked about stimulus perception, discomforts, losing attention, and stimulus sequence patterns. The responses reflected that none of the users perceived tearing, pain, or any discomfort related to stimulation, even in large stimulus sequences (19.7 ± 0.3 min for the four blocks). Also, all stimulation markers with the blue/green color scheme were perceptible in any circumstance. Five of the novice BCI subjects reported failing to perceive some target flashes or getting somnolent, both circumstances at the end of the last block, which probably affected the classification performance, but not the global coherent averages. This somnolence phenomenon was, probably, caused by the copy mode static scenario screen that provides no feedback to users. Consequently, the P300 elicitation and its detection rate were affected. Decision to maintain the same background image and marker distribution in performing the evaluation for all subjects was taken to avoid biases due to these factors. With the evidence that scenario screens are useful, there is confidence for implementing scenario and marker changes as described in Section 2.1. Regarding the stimulus generator developed, no subject reported having perceived target or stimulus patterns. Furthermore, they also reported perceiving the ISI variability that likely caused more expectation. Therefore, our developed target and stimulus sequences generators demonstrated being usable since they were comfortable for users. In addition, while OpenViBE was sufficient for scenario screen evaluation, it currently presents limitations for implementing a free-mode scenario screen session (the actual implementation for navigation is developed over a different architecture).

These results overcame the red/white color scheme used in [41] where four of eight subjects reported some type of discomfort. This red/white scheme was said to present no clearly perceptible stimulus when the markers were placed over bright image areas. A further analysis of the native OpenViBE stimulus generator showed that its sequences always alternated row and column stimulus for square arranged stimulation markers [41]. Furthermore, the scenario screen used a high resolution real image background with the twelve available stimulus perfectly perceivable; furthermore, the stimulation markers' meaning depends on where they were located over the image background.

### 3.2. Global P300 Elicitation

**Figure 6:**
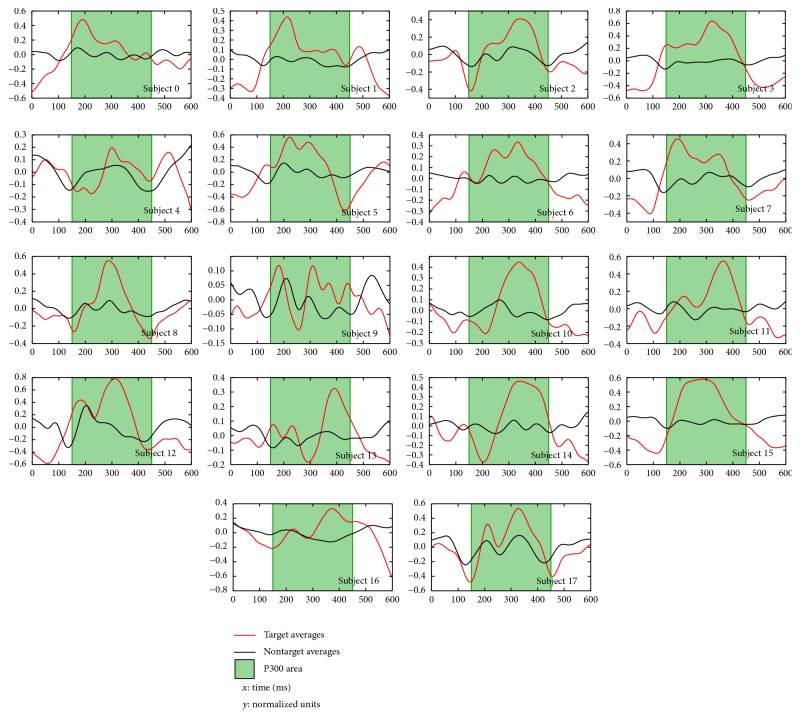
Each subject target (red) and nontarget (black) coherent grand average plots. *X*-axis is the 0–600 ms span, and *Y*-axis is the average amplitude in *μ*V; these averages were computed with *z*-scored epochs (mean: 0 and SD: 1). The green filled areas are the 150–450 ms span.

Paired target and nontarget great coherent averages plots from each of the 18 subjects are shown in Figure 6. One subject was discarded as he slept during two blocks of the experiment. The target averages, in all cases, were different from their respective nontarget averages. 72% of the target averages (subjects 1, 2, 4–8, 10–15, and 17) presented positive peaks within 150–450 ms (Figure 6 green filled area) that are consistent with a P300 elicitation. Furthermore, these peaks are preceded by a negative peak around 200 ms that may be associated with the N200 elicitation. Four subject target averages (0, 3, 6, and 16) had other features not directly associated with expected P300 elicitation, but their respective target and nontarget averages are still different, as is the case for subjects (2, 3, 5, 7, 8, and 17) who had a negative peak within 400–550 ms that might be related to the long latency N400. However, further analysis should be done to identify the meaning of these negative deflections. Those results were consistent with the findings reported by [18, 54, 55]. Finally, subject 9 target average waveform is noisy and does not represent a typical P300 elicitation, yet it is different with respect to its nontarget average counterpart.

The stimulus generator engine developed, particularly, the pseudorandom ISI feature, showed effectiveness on visual steady-state periodic components reduction, as is shown in Figure 6. Except for subject 9, all the coherent grand averages do not have steady-state artifacts taking into account the fact that the preprocessing used a passband 40th FIR filter within 1.5–10.0 Hz; in contrast, when the native OpenViBE stimulus generator was used [41] those filter parameters were 100th and 0.1–2.0 Hz due to the ISI-related components.

Selecting P300 of each subject as the highest peak within 150–450 ms on the target coherent averages (see Figure 6 green areas), all these magnitudes were contrasted with their nontarget analogues by a signed rank Wilcoxon test obtaining a statistical difference (*W* = 0.0; *p* < 0.001) which suggests that attending the target marker generates a different brain response. Furthermore, the median latency of those P300 peaks was 310 (217–337) ms which corresponds to P300 span and is consistent with the results reported in [54–56].

In regard to the statistical analysis of the paired waveforms, it was found that target coherent averages are statistically different from their nontarget pairs (MWUT; *p* < 0.005) considering the 150–450 ms spans, except for the fourth subject. Summarizing, the target perception elicits a response within (150–450) ms differentiable from the nontarget stimulation. To put it differently, the stimulation screen with an image in the background and whose stimulation markers are asymmetrically arranged elicits an adequate response when the subjects perceive the target stimulation in 94% of the cases.

### 3.3. Target Detection in Scenario Screen

#### 3.3.1. Target Detection Performance Analysis over the Subject Sample

**Figure 7:**
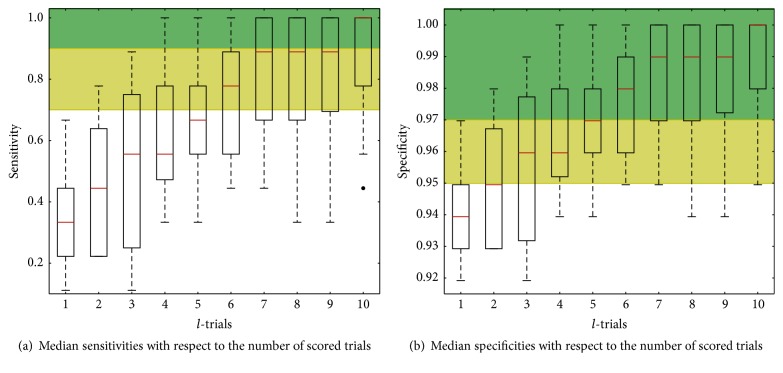
Sensitivity and specificity performances with respect to the number of trials.

Sample median sensitivity, specificity, and accuracy [57, 58] with respect to number of trials (*l*) accumulated for the score calculation are summarized in Table 2. These performance metrics were calculated using the unseen nine repetition data of each subject. More precisely, the sensitivity or true positive rate was the metric related to the proportion of target markers correctly classified. From the 6th scored trial (*l* = 6) the sample median was above 0.75 sensitivity which is the minimum accepted on P300 spelling tasks [2, 4, 6, 8]. Moreover, from the 7th trial (*l* = 7), 0.7 sensitivity was found for 75% of the subjects regardless of the fact that 78% of them had no experience on the task. This is graphically shown in the boxplots of Figure 7(a) where it is seen that the target detection is improved and also the dispersion becomes lower as more trials were scored. This was consistent with the conventional P300 speller knowledge [5, 16, 59], but using a stimulation screen with a snapshot background and no orthographic or semantic stimulation markers.

The performance of five commonly used classifiers for conventional P300 spelling task (LASSO-LDA, shrinkage-LDA, Linear-SVM, Radial Basis Function SVM, and SWLDA) were evaluated for the scenario screen [60]. For this study all the hyperparameters of each classifier were appropriately optimized. No statistical differences were found among the five classifier performances but LASSO-LDA presented a lower variance nevertheless. Thus, only LASSO-LDA was utilized in this work; a reduction from 4 to 2 in the decimation factor with respect to [60] was implemented reflected in lower variances in the subject sample sensitivity as Figure 7 shows. Additionally, partial coherent averages as feature vectors and linear-SVM were tested for P300 detection [41], although the sensitivity was at least 0.7 in most of the cases using 10 trials; the target marker detection rates shown in Table 2 are higher even from five scored trials (*l* = 5).

In terms of specificity or true negative rate which was the metric related to the proportion of well labeled nontargets shown in Table 2, the median specificity was higher than 0.97 from *l* = 5 which means that 3 false targets were detected.  Figure 7(b) shows the corresponding specificity boxplots with respect to the number of scored trials. From *l* = 7, 75% of the subjects got at most 3 misclassified nontargets. Table 2 also shows the accuracies which might be considered good given that they are always higher than 0.85; however, this is a biased measure given the unbalanced number of target and nontarget feature vectors. For this reason we prefer measuring the performance in terms of sensitivity and specificity. Additionally, when ten scores are accumulated (*l* = 10) the average time to perform a detection is 40 s (12 × 10 × [.125 + .17] + 9 × 0.5); these values correspond to the number of available target markers, number of trials, IS, average ISI, number of ITI, and ITI.

On the other hand, the summarized performances of the screens with image backgrounds reported in [30, 31] suggested that a row/column stimulation mode was feasible for symmetrically arranged stimulation markers. However, when this stimulation mode was used in a scenario screen [41], a minimum distance constraint among stimulation markers was used to avoid peripheral stimulus perception of nontargets. In this work, that constraint was no longer necessary because of the single marker mode usage and the high performances obtained.

In sum, the scenario screen was capable of eliciting detectable P300 through LASSO-LDA with performances higher than 0.75 from *l* = 6. The highest median performances were obtained accumulating all the trails (*l* = 10), with 1.0 (0.78–1.00) and 1.0 (0.98–1.00) being the median sensitivity and specificity, respectively. Thus, the scenario screen resulted in a very suitable P300 speller variation for selecting the path for a wheelchair, even when more than 78% of the subjects had no experience in P300 spelling tasks. These results were consistent with [5, 18, 28, 29], even when the scenario screen stimulation markers did not use an orthographic or semantic scheme; thus the implicit findings reported in [30, 31] in terms of stimulation screen evaluation and target marker detection were also complemented.

#### 3.3.2. Individual Analysis of the Target Detection Performances

**Figure 8:**
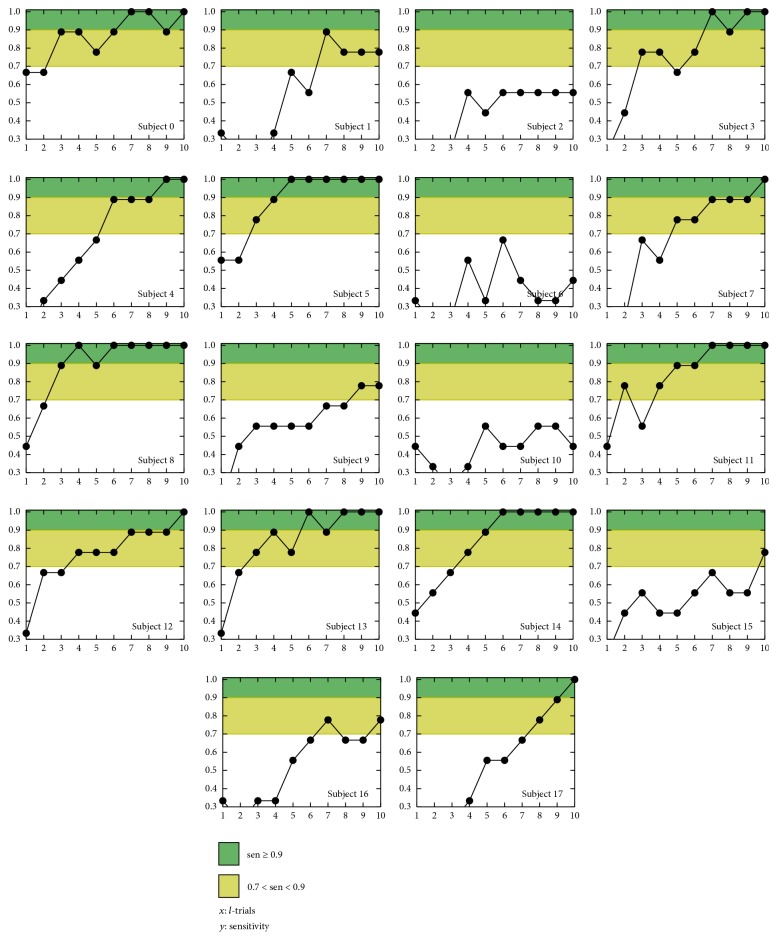
Individual sensitivities with respect to the number of trials scored *l* = {1,…, 10}. Green filled area represents sensitivities greater than 0.9, while yellow areas represent those within [0.7,0.9.

Taking into account the 44% of the subjects (0,3, 4,5, 8,11,13,14) who reached a plateau with at least two 1.0 sensitivity points (see Figure 8), the median sensitivities for *l* equal to five, six, and seven are, respectively, 0.78 (0.64–0.81), 0.94 (0.89–1.00), and 1.00 (0.97–1.00) which are high performances even with *l* = 5. For all these subjects the number of trials might be reduced to seven with no performance cost; this repetition shortening would imply a 27.5% time reduction. Moreover 28% of subjects reached high performances with seven scored trials, despite their inexperience on P300 spelling tasks. On the other hand, the 39% of the subjects (1,7, 9,12,15,16,17) that had not reached a plateau, got a median sensitivity of 0.78 (0.67–0.89) with seven trials scored; furthermore, the lower quartile included the minimum performance for spelling tasks that was consistent with the findings reported in [5, 18, 28, 29].

**Figure 9:**
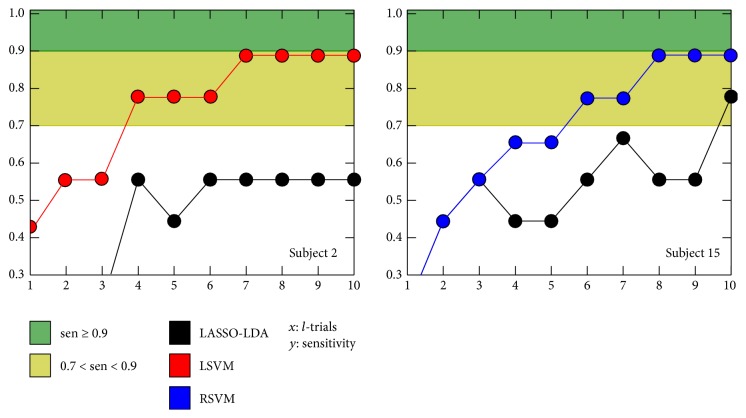
Sensitivity with respect the number of scored trials. The black line is LASSO-LDA, while red and blue ones correspond to LSVM and RSVM, respectively.

In regard to the three lowest sensitivity performances (subjects 2, 6, and 10) whose average was 0.48 (SD = 0.1), all these subjects presented a performance consistently low despite the number of scored trials. Although this behavior might be associated with subjects' wrongly executed task, when LASSO-LDA was substituted by a Linear Support Vector Machine (Linear-SVM) for subject 2, the detection rate had improved up to 57% in terms of sensitivity. That behavior suggested that a customized classifier selection is likely to improve the detection rates [54, 55, 60]. A similar case was subject 15 whose performance had improved when a Radial Basis Function SVM was utilized. A whole behavior comparison between LASSO-LDA and the best classifier of subjects 2 and 15 with respect to the number of scored trials is shown in Figure 9. Notwithstanding other four classifiers (Linear-SVM, Radial Basis Function SVM, Shrinkage-LDA, and SWLDA) tested in subjects 6 and 10, no sensitivity improvement was reached. However as Figure 8 shows subjects 6 and 10 had sensitivities of 0.67 and 0.56 with six and eight scored trials, respectively. These results reinforced the necessity of an exhaustive customization of the pattern recognition method.

**Figure 10:**
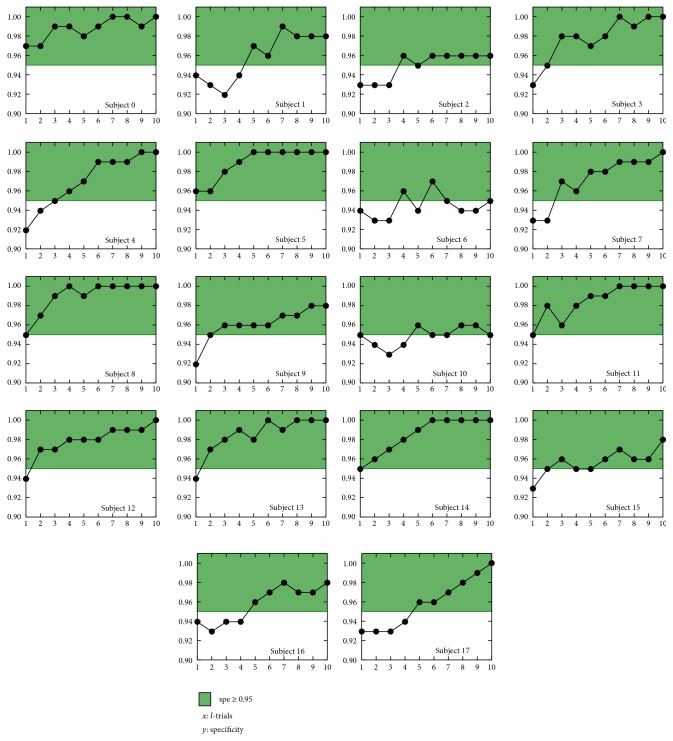
Individual specificities with respect to the number of trials scored *l* = {1,…, 10}. Green filled area represents specificities greater than 0.95.

Performing a similar analysis for the specificity, the results obtained for all subjects were high, always greater than 0.92, which implied that only a small amount of nontargets were labeled as targets (Figure 10 and Table 2). Median misclassified number of targets for *l* = 10 was 1.5 (1.0–3.75); this result, on the scenario screen, meant that the destination was not correct.

When ten scored trials were considered, 11 of 18 subjects (61%) accomplished the correct selection of 100% of the targets, despite eight of them (44%) having no previous experience on P300 spelling task. This is summarized in Table 3. Additionally, four subjects (22%) detected correctly seven of nine targets while three subjects (17%) detected between four and five. However, when LSVM and RSVM are, respectively, used for subjects 2 and 15, there was a global correct target improvement due to 72% of the subjects having a sensitivity higher than 0.87. In contrast, only subjects 6 and 10 (11%) had four of nine correctly detected targets. Although those two subjects presented a target coherent grand average with P300 features and their target and nontarget averages were statistically different, none reached the minimum performance. Yet, subject 4 had no statistical differences between his coherent averages; he reached 1.0 in both sensitivity and specificity. Furthermore, subject 9 (see Figure 6) had a target coherent average with no P300 features but he was capable of correctly selecting seven of nine trials nevertheless, therefore reaching more than the minimum performances for the tasks. In conclusion, those four cases showed that a global P300 elicitation was not necessarily related to the target detection performance. In regard to accuracy, metric defined in [53], Tables 2 and 3 show accuracies higher than 0.87 from single trial (*l* = 1); additionally, the AUROC with ten trials (*l* = 10) is higher than 0.87 ± 0.07 for all subjects.

All recordings were performed in realistic conditions with no sound isolation; therefore, subjects were prone to listen to distracting noises, for instance, voices, fan noise, alarms, or phones. Yet, 89% of the subjects accomplished the correct selection of more than seven targets (sensitivity > 0.75) and discriminated at least 97 nontargets (specificity > 0.97). Furthermore, 12 of the 16 highest performances (75%) were obtained by unexperienced subjects. Hence the P300 stimulation screen variation with an image background of asymmetrically arranged stimulation markers seems reliable for a contextual and real-time generated stimulation screen for commanding a robotic navigation system of a wheelchair using destination-based stimulus sequences.

## 4. Conclusions

A stimulation screen with an image background whose stimulation markers were asymmetrically arranged and a stimulation sequence generator were developed and evaluated. Using this stimulator, target coherent grand averages were statistically different from their corresponding nontarget averages, except for one subject, who nonetheless reached high sensitivity and specificity. It was also corroborated that target and nontarget averages within 150–450 ms are statistically different.

The scenario screen and its stimulus sequencer showed that they are feasible for use on the wheelchair navigation task for destination-based sequences given that 89% of subjects were able to select the target with high performances. Regardless of the fact that 78% of the subjects had no previous experience in using BCI systems, 72% of the sample reached high sensitivity and specificity performances. In addition, only two unexperienced subjects had performances lower than 0.7. LASSO-LDA altogether with the voting scheme was able to detect the target markers with high performances in almost all subjects. Further analysis of LASSO feature selection is needed to explain why in some cases a direct relationship between the P300 global elicitation and the correct target detection rates cannot be established. Notwithstanding the fact that LASSO-LDA approach had the highest performances on 89% of the subjects, an individualized classifier method might still be considered.

The occasional occurrence of clusters of stimulation markers on a particular scenario screen might contribute to false target detection due to the target and nontargets nearness. An error analysis of those clusters is required in order to verify spatial correlations between the misclassified targets and the true targets. More importantly, the misclassified targets might not have a high impact on the wheelchair navigation task when they are near the true targets; the navigation route and final wheelchair position will be close to the desired one. That is to say, misclassified targets might have lower impact on the navigation accomplishment than their misclassified counterparts on the conventional P300 spelling task where a mistake distorts the message orthographically or semantically [5, 11, 14, 18, 25, 29, 33, 54].

Although all the recordings were performed under realistic conditions, high target and nontarget detection performances were achieved on most subjects. Causes that might impact the performances were task misunderstanding and confusion with the scenario screen usage due to its eight markers with the same character or due to loss of attention. Only two subjects had performances lower than 0.7 sensitivity. These two subjects were able to select correctly 4 of 9 targets which are not directly associated with a random selection given the amount of data (number of epochs and stimulation markers) to be processed to get a target.

Despite the fact that almost all subjects had no discomfort in using the scenario screen, some of them reported somnolence and failing to perceive some targets on the fourth block. This somnolence might be linked to no provision of feedback to the user. This is in accordance with results of other works that relate the ERP elicitation to user motivation [39, 61]. In regard to the stimulation screen features, a blue/green stimuli color scheme seems the correct election since no subject reports discomfort related to stimulus attention, in contrast to the previous reported red/white scheme [41].

Although Escolano et al. [30, 31] reported two stimulation screens with image background, those were implemented differently from scenario screen as described in Introduction. Nonetheless, both reports had an entirely distinct aim with respect to this paper; that is, they were focused on the evaluation of navigation not on the stimulation screen evaluation nor the stimulus presentation features that improve the target detection.

Finally, the stimulus sequence generator developed substitutes the native OpenViBE sequencer overcoming its limitations at implementing the single marker stimulus mode. Other OpenViBE inconveniences, which did not compromise the realization of the evaluations reported herein, indicate that other software architectures should be used for a release version of the navigation control.

## Figures and Tables

**Table 1:** Comparison among several works where P300 speller variants were used to generate commands for a wheelchair navigation control system. *t–nt diff*: target–nontarget difference. ^*∗*^Included due to the asymmetric markers arrangement.

	Stimulation	Background	Markers	P300 analysis
	*Color scheme*	*Type*	*Number*	*# subjects*
	*Mode*	*Color depth*	*Encoding*	*Latency*
	*Presentation*		*Arrangement*	*t*-*nt diff.*
Scenario screen (this work)	Blue/green	Real image	12	18
	Single marker	24-bit	Unicode	Yes
	Destination-based and fixed displacements		Asymmetrical	Yes

Escolano [30, 31]	White-green/blue	Real image	12	2
	Row/column	Gray scale and 24-bit	Icon-images	No, yes
	Fixed displacements		Symmetrical	No

Iturrate [32]	Black/blue/white	Virtual image	20	5
	Row/column	8-bit	Icon-images	No
	Fixed displacements		Symmetrical	No

Gentiletti [33]	Gray/white	Solid black	12	2
	Row/column	5-bit	Images + text	No
	Fixed displacements		Symmetrical	No

Lopes [34]	Gray/green	Solid black;	7	11
	Single marker	5-bit	Arrows + text	No
	Fixed displacements		Symmetrical	No

Rebsamen [35, 36]	Blue/white	Solid gray	7	5
	Single marker	5-bit	Box + text	No
	Destination-based		Symmetrical	Yes

Ganin [28, 29]^*∗*^	Image/white	Solid gray	9	14
	Single marker	24-bit	Image + text	Yes
	Fixed displacements		Asymmetrical	Yes

**Table 2:** Subject sample median sensitivities, specificities, and accuracies with respect to the number of trials scored *l* = {1,…, 10}. IQR: interquartile range.

*l* trials	Sensitivity (IQR)	Specificity (IQR)	Accuracy (IQR)
(1)	0.33 (0.22–0.44)	0.94 (0.93–0.95)	0.89 (0.87–0.91)
(2)	0.44 (0.22–0.64)	0.95 (0.93–0.97)	0.91 (0.87–0.94)
(3)	0.56 (0.25–0.75)	0.96 (0.93–0.98)	0.93 (0.88–0.96)
(4)	0.56 (0.47–0.78)	0.96 (0.95–0.98)	0.93 (0.91–0.96)
(5)	0.67 (0.56–0.78)	0.97 (0.96–0.98)	0.94 (0.93–0.96)
(6)	0.78 (0.56–0.89)	0.98 (0.96–0.99)	0.96 (0.93–0.98)
(7)	0.89 (0.67–1.00)	0.99 (0.97–1.00)	0.98 (0.94–1.00)
(8)	0.89 (0.67–1.00)	0.99 (0.97–1.00)	0.98 (0.94–1.00)
(9)	0.89 (0.69–1.00)	0.99 (0.97–1.00)	0.98 (0.95–1.00)
(10)	1.00 (0.78–1.00)	1.00 (0.98–1.00)	1.00 (0.96–1.00)

**Table 3:** Individual sensitivity, specificity, and accuracy achieved when ten trials are scored (*l* = 10). *j* and *k* are, respectively, the number of correctly classified targets and nontargets. Meanwhile, the correctly labeled target and nontarget count is *m*. AUROC: area under the receiver operating characteristic curve [37]. SD: standard deviation.

Subject	Sensitivity (*j*-correct)	Specificity (*k*-correct)	Mean AUROC (SD)	Accuracy (*m*-correct)
17	1.00 (9)	1.00 (99)	0.98 (0.02)	1.00 (108)
11	1.00 (9)	1.00 (99)	0.99 (0.01)	1.00 (108)
3	1.00 (9)	1.00 (99)	1.00 (0.00)	1.00 (108)
4	1.00 (9)	1.00 (99)	1.00 (0.01)	1.00 (108)
5	1.00 (9)	1.00 (99)	1.00 (0.00)	1.00 (108)
7	1.00 (9)	1.00 (99)	0.98 (0.02)	1.00 (108)
8	1.00 (9)	1.00 (99)	1.00 (0.01)	1.00 (108)
12	1.00 (9)	1.00 (99)	1.00 (0.00)	1.00 (108)
13	1.00 (9)	1.00 (99)	0.85 (0.09)	1.00 (108)
14	1.00 (9)	1.00 (99)	0.99 (0.01)	1.00 (108)
0	1.00 (9)	1.00 (99)	0.96 (0.05)	1.00 (108)
9	0.78 (7)	0.98 (97)	0.95 (0.06)	0.96 (104)
16	0.78 (7)	0.98 (97)	0.91 (0.05)	0.96 (104)
15	0.78 (7)	0.98 (97)	0.95 (0.03)	0.96 (104)
1	0.78 (7)	0.98 (97)	0.86 (0.06)	0.96 (104)
2	0.56 (5)	0.96 (95)	0.92 (0.04)	0.93 (100)
10	0.44 (4)	0.95 (94)	0.87 (0.06)	0.91 (98)
6	0.44 (4)	0.95 (94)	0.87 (0.07)	0.91 (98)
